# Therapeutic strategies targeting mechanisms of macrophages in diabetic heart disease

**DOI:** 10.1186/s12933-024-02273-4

**Published:** 2024-05-15

**Authors:** Chaoyue Zhang, Yunke Shi, Changzhi Liu, Shivon Mirza Sudesh, Zhao Hu, Pengyang Li, Qi Liu, Yiming Ma, Ao Shi, Hongyan Cai

**Affiliations:** 1 Cardiovascular Clinical Medical Center, The First Affiliated Hospital of Kunming Medical University, Kunming, China; 2 Department of Cardiovascular Surgery, The First Affiliated Hospital of Kunming Medical University, Kunming, China; 3 Faculty of Medicine, St. George University of London, London, UK; 4 University of Nicosia Medical School, University of Nicosia, Nicosia, Cyprus; 5 Department of Geriatric Cardiology, The First Affiliated Hospital of Kunming Medical University, Kunming, China; 6 Division of Cardiology, Pauley Heart Center, Virginia Commonwealth University, Richmond, VA USA; 7 Wafic Said Molecular Cardiology Research Laboratory, The Texas Heart Institute, Houston, TX USA

**Keywords:** Diabetic heart disease, Diabetic cardiomyopathy, Macrophages, Drug therapy

## Abstract

Diabetic heart disease (DHD) is a serious complication in patients with diabetes. Despite numerous studies on the pathogenic mechanisms and therapeutic targets of DHD, effective means of prevention and treatment are still lacking. The pathogenic mechanisms of DHD include cardiac inflammation, insulin resistance, myocardial fibrosis, and oxidative stress. Macrophages, the primary cells of the human innate immune system, contribute significantly to these pathological processes, playing an important role in human disease and health. Therefore, drugs targeting macrophages hold great promise for the treatment of DHD. In this review, we examine how macrophages contribute to the development of DHD and which drugs could potentially be used to target macrophages in the treatment of DHD.

## Introduction

Diabetes mellitus (DM) is a widespread condition with costly treatment. In 2045, DM is projected to affect 783 million individuals worldwide and incur an annual economic expense of 1054 billion USD [1]. Type 2 DM (T2DM) accounts for 90–95% of all diabetes cases [2], and people with diabetes have more than twice the risk of developing heart failure (HF) than do people without diabetes [3, 4]. DM and its complications are a serious threat to human health and have become a global public health problem [5]. The pathologic mechanisms of different diabetic complications have a high degree of commonality at the vascular level.

One of the most common forms of diabetic heart disease (DHD) is diabetic cardiomyopathy (DCM). Although researchers have devoted significant resources toward developing a treatment for DCM, only a handful of possibilities have emerged, such as using sodium-glucose cotransporter 2 inhibitors (SGLT2is) [6], glucagon-like peptide 1 receptor agonists (GLP1RAs) [7], and metformin [8]. Unfortunately, these treatments have not significantly improved patient prognosis. A central pathogenic mechanism of DHD is the inflammatory response, which exacerbates oxidative stress and endoplasmic reticulum stress [9]. Macrophages, which execute the inflammatory response, influence the course of DHD through the secretion of various inflammatory factors [10]. Therefore, a drug therapy targeting macrophages may be a promising new direction for improved DHD treatment.

Type 1 DM (T1DM) and T2DM, the two primary forms of DM, have different pathogenic mechanisms. T1DM is mainly caused by the autoimmune destruction of pancreatic β-cells and occurs in younger patients, whereas T2DM is caused by insulin resistance and relative insulin deficiency and is usually seen in older and overweight patients. Both T1DM and T2DM can induce DHD and impaired cardiac function. They share some clinical presentations (e.g., diastolic dysfunction, systolic dysfunction, HF) and pathogenic mechanisms (e.g., cardiac hypertrophy and fibrosis, oxidative stress, inflammation, metabolic dysfunction) [11]. Patients with T2DM are more likely to experience ventricular diastolic dysfunction compared to those with T1DM [12]. Although macrophages are involved in DHD, their specific roles in T1DM- and T2DM-induced DHD and possible ways of therapeutic modulation have not been well studied. Common clinical pharmacological treatments, such as with SGLT2is and GLP1RAs, have not been studied in cardiovascular outcomes trials in patients with T1DM, despite studies showing that these treatments can lower hemoglobin A1c (HbA1c) levels, reduce body weight, facilitate glucose control, and reduce insulin dose requirements [13–15]. In the present review, we discuss the role of macrophages in DHD and their pharmacological targeting, with a focus on T2DM-related DHD.

## Overview of macrophages: origin, polarization, and function

**Fig. 1:**
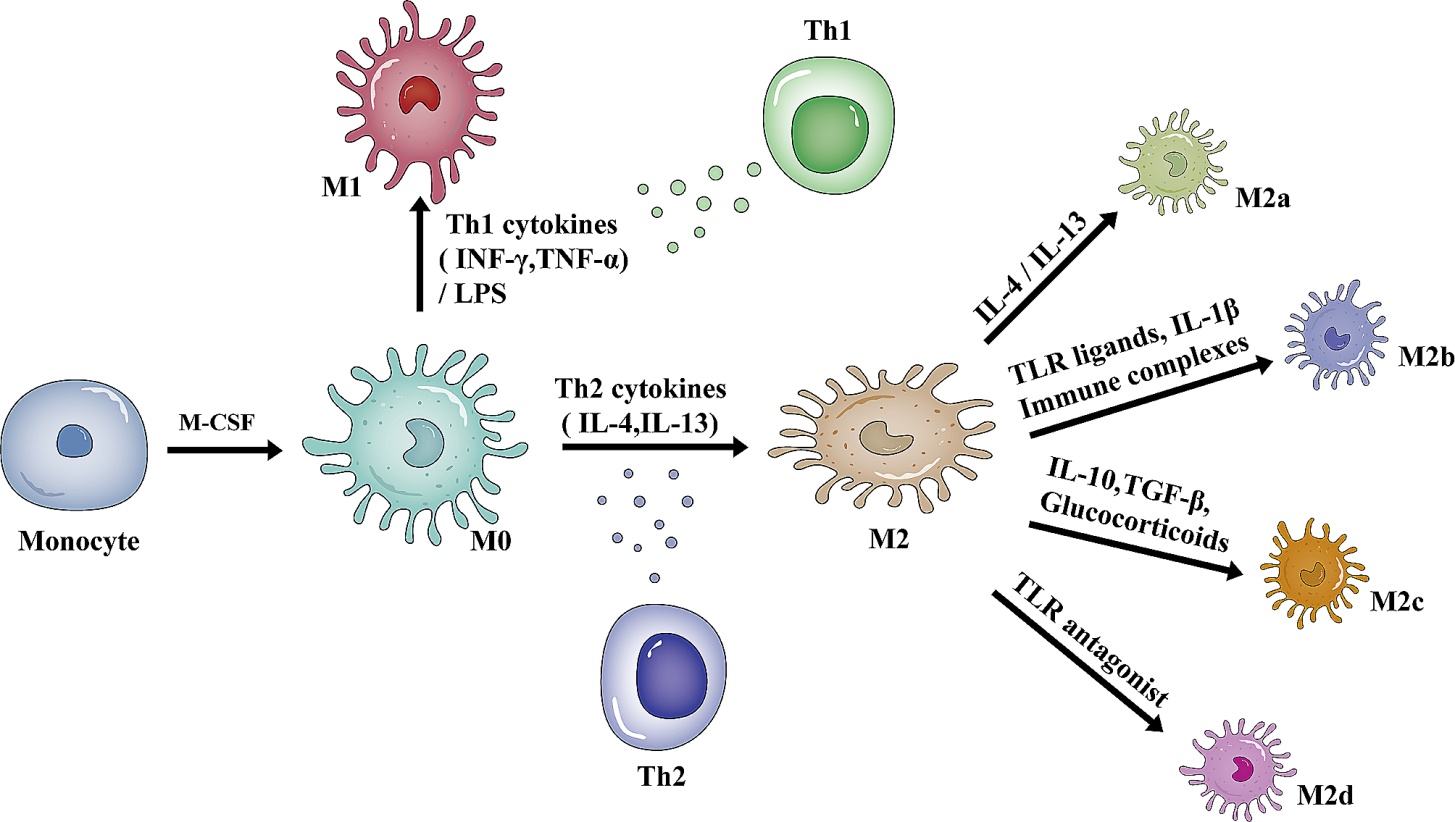
A schematic representation of macrophage polarization. Monocytes can be differentiated into M0 macrophages in response to M-CSF stimulation. M0 macrophages can be polarized into M1 macrophages in response to LPS or Th1 cytokines (INF-γ, TNF-α). M0 macrophages are polarized into M2 macrophages in response to Th2 cytokines (IL-4, IL-13). Depending on the stimuli, M2 macrophages are subdivided into M2a, M2b, M2c, and M2d macrophages. Abbreviations: M-CSF: macrophage colony-stimulating factor, INF-γ: interferon-γ, TNF-α: tumor necrosis factor-α, LPS: lipopolysaccharide, IL: interleukin, TGF-β: transforming growth factor-β, TLR: toll-like receptor

Macrophages are evolutionarily conserved innate immune cells that play an essential role in human health and disease [16]. Macrophages are heterogeneous, with phenotypes and functions that are regulated by the surrounding microenvironment. Macrophage polarization is the process by which macrophages show specific phenotypic and functional responses upon microenvironmental stimulation and signaling [17]. M0 macrophages are induced by macrophage colony-stimulating factor (M-CSF) to differentiate from monocytes and represent the initial state of macrophage polarization [18]. Depending on the type of stimulus and the pattern and functional properties of surface molecules and secreted cytokines, M0 macrophages polarize into one of two main subpopulations: M1 and M2 macrophages [19]. An illustration of macrophage polarization is shown in Fig. 1. M1 macrophages have potent anti-microbial and anti-tumor activities and participate in the clearance of pathogens by activating nicotinamide adenine dinucleotide phosphate oxidase (NOX) and producing reactive oxygen species (ROS) during injury or infection [20]. Alternately activated or M2 macrophages, which have anti-inflammatory and immunomodulatory effects, are polarized by T helper 2 (Th2) cytokines interleukin-4 (IL-4) and IL-13 [21–23]. M2 macrophages have a strong phagocytic ability, can remove dead cells, promote tissue repair and wound healing, and have pro-angiogenic and profibrotic properties [24].

M1 and M2 macrophages are not two distinct cell populations [25] but are present in a spectrum of different functional states, including pro-inflammatory and anti-inflammatory phenotypes of the cells [26]. The concept of M1/M2 is based on several in vitro studies in which macrophages were stimulated in culture with a defined set of factors [27]. Compared to in vivo situations, macrophages cultured outside their native tissue microenvironment have significant differences in their polarization and function [28]. Given the complexity of M1/M2 polarization [27], the functional and stimulant-based understanding of macrophages in the context of DHD warrants further exploration.

### The role of macrophages in diabetic heart disease

**Fig. 2:**
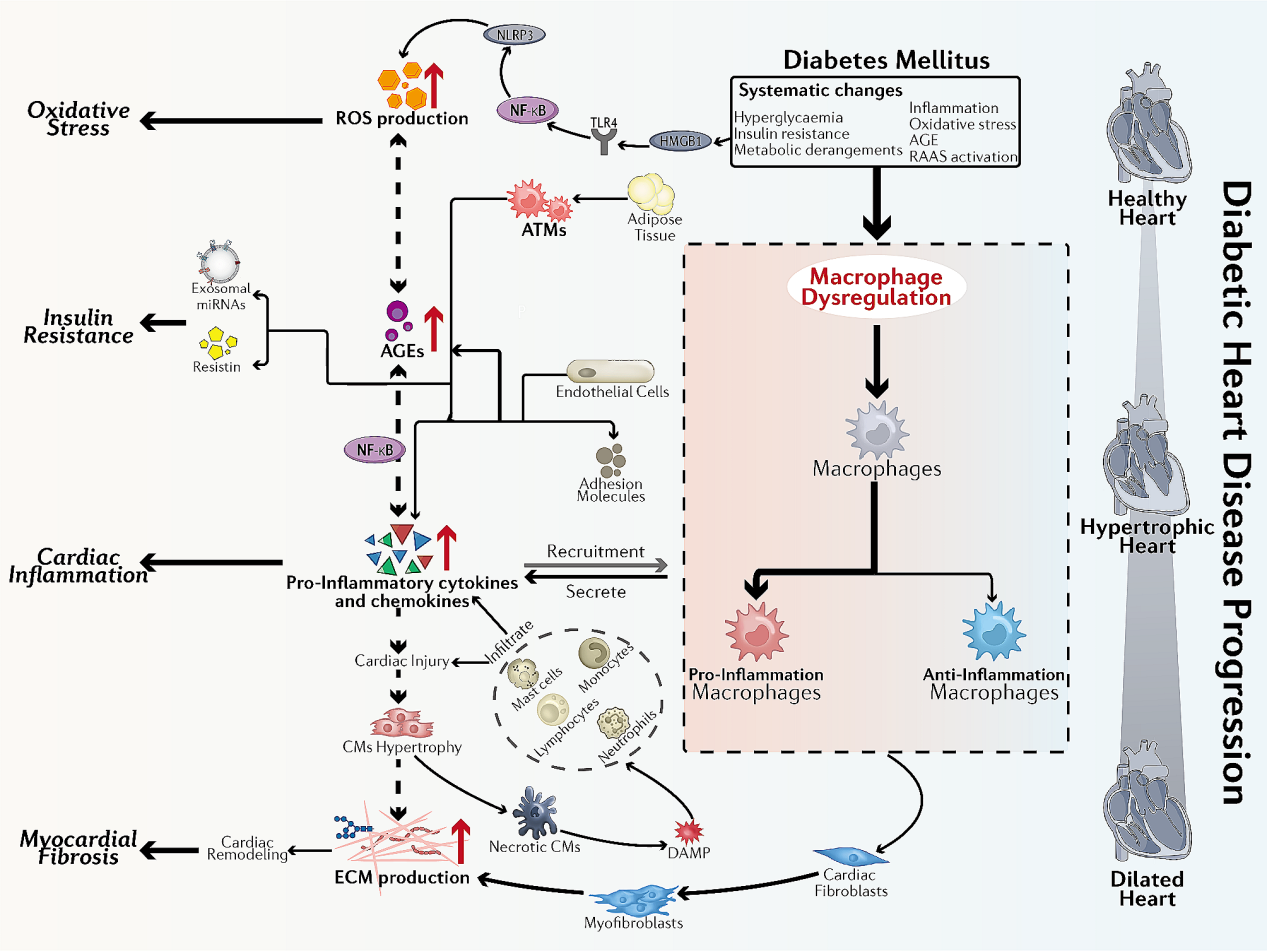
Schematic diagram of the mechanisms involved in the development of DHD by macrophages. The hyperglycemic environment in diabetic patients induces CM injury, and necrotic CMs activate mast cells by releasing DAMPs, which induce the secretion of TNF-α and IL-1β, leading to the activation of ECs. Activated ECs induce monocytes to differentiate into CCR2+ macrophages and CCR2-macrophages; the former create an inflammatory environment and remove necrotic tissue, while the latter release IL-10 and TGF-β1, which activate fibroblasts to differentiate into myofibroblasts and produce ECM and collagen for tissue repair. The hyperglycemic environment also directly activates ECs, which secrete cytokines and chemokines that induce macrophage polarization to the M1 phenotype. In turn, M1 macrophages secrete inflammatory factors that promote cardiac inflammatory responses and insulin resistance. In addition, a hyperglycemic environment leads to increased production of AGEs, and the overproduction of AGEs promotes ROS production. The hyperglycemic environment can also act on HMGB1, gradually activating their downstream reactions, leading to increased ROS generation, and exacerbating oxidative stress. Furthermore, ATMs can secrete miRNA-containing exosomes that act on insulin-target cells to develop insulin resistance. Abbreviations: CM: cardiomyocyte, DAMP: danger-associated molecular pattern, TNF-α: tumor necrosis factor-α, IL: interleukin, EC: endothelial cell, TGF-β1: transforming growth factor-β1, ECM: extracellular matrix, AGE: advanced glycation end product, ROS: reactive oxygen species, HMGB1: high mobility group box 1, ATM: adipose tissue macrophage, NF-κB: nuclear factor kappa-B, NLRP3: NLR family pyrin domain-containing protein 3, TLR4: toll-like receptor 4

According to the currently accepted pathophysiologic mechanisms of DHD [29–32], insulin resistance and metabolic derangements induce hyperglycemic environments in patients with DM [33]. As the disease progresses, the microenvironment promotes inflammatory responses, oxidative stress, advanced glycation end product (AGE) production, and renin-angiotensin-aldosterone system (RAAS) activation. These pathophysiologic abnormalities promote cardiac stiffness, hypertrophy, and fibrosis, leading to diastolic dysfunction, systolic dysfunction, and HF. Below, we discuss the role of macrophages in these pathophysiologic mechanisms, as illustrated in Fig. 2.

### Cardiac inflammation

DM is characterized by hyperglycemia and insulin resistance, which promote an inflammatory state in the body [9]. The inflammatory response is a critical mechanism by which the heart responds to injury and undergoes adaptive remodeling [34]. The hyperglycemic environment in patients with DM affects protein glycation and the production of AGEs, which activate the nuclear factor kappa B (NFκB) pathway in macrophages. This activation, in turn, induces the production of inflammatory cytokines, leading to the activation of macrophages toward an inflammatory phenotype [35–37]. The dysregulation of macrophages between pro-inflammatory and anti-inflammatory phenotypes promotes excessive inflammation and cardiac injury [38]. In individuals with diabetes, macrophages secrete cytokines and chemokines that play a role in developing cardiomyocyte hypertrophy and extracellular matrix (ECM) remodeling. Stimulated by prolonged hyperglycemia, pro-inflammatory macrophages and lymphocytes accumulate and infiltrate into the heart, promoting the secretion of cytokines, such as tumor necrosis factor (TNF), IL-6, IL-1β, interferon-γ (INF-γ), and transforming growth factor-β (TGF-β), which can induce or exacerbate cardiac injury, leading to further adverse remodeling and myocardial fibrosis [39–42].

High mobility group box 1 (HMGB1) is a nuclear chromatin protein that interacts with nucleosomes, transcription factors, and histones to promote the transcription of many genes associated with inflammatory processes [43, 44]. More precisely, in a hyperglycemic environment, HMGB1 can activate toll-like receptor 4 (TLR4) [45]. TLR4 is expressed in cardiac inflammatory cells, cardiac fibroblasts, and cardiomyocytes [46], and its role in mediating inflammatory signaling in the development of DHD has been demonstrated in animal models [47–50].

### Insulin resistance

Inflammation can lead to the development of insulin resistance. Studies have shown that inhibiting inflammatory response pathways can greatly enhance insulin sensitivity in both animals and humans [51, 52]. TNF-α and monocyte chemoattractant protein 1 (MCP1) play important roles in this process. TNF-α is involved in insulin resistance by activating inflammatory kinases, including c-Jun N-terminal kinase (JNK) [53] and inhibitor kappa B kinase (IKK) [54]. Previous studies have shown that increased TNF-α and macrophage levels in the adipose tissue (AT) of obese mice and humans are associated with insulin resistance [55–57]. Macrophages, especially adipose tissue macrophages (ATMs), which are a significant source of pro-inflammatory cytokines, contribute to reduced insulin sensitivity in a paracrine and potentially endocrine manner [58]. Reduction of ATMs or TNF-α by their depletion or ablation of MCP1 or its receptor in mice was associated with improved insulin sensitivity [59–61]. In addition, hepatic insulin resistance is observed with increased TNF-α expression in AT when macrophages are recruited to AT by the overexpression of MCP1. Moreover, knockout of MCP1 protected against high-fat-diet (HFD)-induced insulin resistance [60].

ATMs can also affect insulin sensitivity and glucose homeostasis via non-inflammatory pathways. Obesity triggers lysosome biogenesis, resulting in lipid catabolism and buildup of ATMs, specifically of CD11c+ macrophages [62] or Trem2+ macrophages [63]. Deficiency of Trem2 in mice on HFD reduces lipid accumulation in Trem2+ ATMs. These macrophages contribute to aggravated glucose intolerance [63]. Consistently, lipid storage in ATMs can regulate systemic glucose homeostasis and insulin sensitivity [64].

### Myocardial fibrosis

Cardiac fibrosis is an unavoidable consequence of chronic myocardial injury and is characterized by the accumulation of ECM proteins in the cardiac interstitium [65]. Intracellular signaling and crosstalk between resident cardiac macrophages and other cells play a crucial role in the onset, propagation, and progression of cardiac inflammation, tissue remodeling, and myocardial fibrosis [66]. The two main types of resident cardiac macrophages are C-C motif chemokine receptor 2 (CCR2)+ and CCR2- macrophages. CCR2+ macrophages maintain their numbers primarily through monocyte recruitment and are mainly involved in pro-inflammatory responses, whereas CCR2- macrophages are primarily embryonic in origin and play a role in promoting angiogenesis and tissue repair [67]. Early in myocardial injury, cardiomyocytes undergo necrosis and release endogenous danger-associated molecular patterns (DAMPs). DAMPs activate mast cells to release pro-inflammatory cytokines, such as TNF-α and IL-1β, as well as chemokines, which activate endothelial cells and recruit activated CCR2+ monocytes and neutrophils. CCR2+ monocytes differentiate into macrophages at the site of injury and work with neutrophils to create an inflammatory environment and remove necrotic tissue [68]. In contrast to CCR2 + macrophage function, CCR2- macrophages are activated in response to myocardial injury and release IL-10 and TGF-β1 [65], which activate fibroblasts to differentiate into myofibroblasts that produce ECM and collagen for tissue repair. However, cardiomyocyte death and increased cardiac inflammation lead to enhanced activation of myofibroblasts and uncontrolled production of ECM and collagen, which accumulate in damaged cardiac tissue and eventually lead to myocardial fibrosis [69, 70].

In addition, the imbalance of M1 and M2 macrophages may be an important factor in promoting myocardial fibrosis. In the context of DHD, AGEs have the ability to direct macrophages toward a pro-inflammatory M1 phenotype, which leads to myocardial injury and, ultimately, myocardial fibrosis [71].

### Oxidative stress

Oxidative stress occurs from an imbalance in the generation of free radicals and antioxidants [72]. The overproduction of ROS is believed to be a central mechanism of cardiac inflammation and remodeling [73, 74] and contributes to oxidative stress in both the early and late stages of DCM [75, 76]. In a hyperglycemic environment, HMGB1 can activate TLR4 [45]. Activation of TLR4 leads to the activation of NFκB and the NLR family pyrin domain-containing protein 3 (NLRP3) inflammasome, which recruits procaspase-1 [77, 78]. Activated caspase-1 serves as an enhancer of multiple pro-inflammatory pathways involving NFκB, chemokines, and ROS. An NFκB positive-feedback loop further increases NLRP3 inflammasome assembly and procaspase-1 activation, which stimulates additional ROS production [78].

Another key enzyme in ROS production is NOX [79]. In the diabetic heart, hyperglycemia leads to aberrant activation of mitochondrial NOX, which exacerbates ROS production [80]. In addition, macrophages under hyperglycemic conditions have reduced glyceraldehyde-3-phosphate dehydrogenase (GAPDH) activity, leading to the increased formation of AGEs and their precursors [81]. AGEs can be released extracellularly and bind to AGE receptors (RAGEs) in an autocrine and paracrine manner, resulting in increased ROS production [82]. The interaction between AGEs and RAGEs also promotes M1 polarization by inducing the secretion of IL-6 and TNF-α, which further exacerbates ROS production [83].

### Trained immunity

Both T1DM and T2DM are characterized by hyperglycemia, and the primary focus of treatment is to lower blood glucose levels. However, some studies have found that the risk of cardiovascular complications persists in some diabetic patients even after glucose-lowering therapy [84]. This phenomenon is referred to as a “legacy effect” or “metabolic memory” [85]. Innate myeloid cells, such as macrophages and monocytes, have the potential for enhanced responsiveness to secondary stimulation, a phenomenon referred to as “trained immunity” [86]. Trained immunity is the process by which innate immune cells undergo long-term functional reprogramming after brief exposure to a stimulus, which persists after removing that stimulus [87]. The “legacy effect” may be related to this “trained immunity” of macrophages. Edgar et al. [88] found that bone marrow-derived macrophages (BMDMs) cultured in a high-glucose environment had increased glycolysis and enhanced polarization toward the M1 phenotype. The BMDMs isolated from diabetic mice, when cultured at physiological glucose concentrations, had persistent pro-inflammatory status despite normalization of the external glucose concentration. Therefore, therapies that target macrophage-trained immunity may offer a potential approach to treating DHD.

### Pharmacological targeting of macrophages in DHD

DHD includes both coronary artery disease (CAD) and DCM. Therapeutic strategies for CAD are well established, whereas therapeutic measures for DCM, in sharp contrast, are lacking. The plasticity and adaptability of macrophages in response to various stimuli make them attractive targets for pharmacotherapy. Next, we will summarize several drugs that modulate macrophage function and have the potential to treat DHD.

### Sodium-glucose cotransporter 2 inhibitors (SGLT2is)

Sodium-glucose cotransporters (SGLTs) promote the reabsorption of blood glucose after glomerular filtration in the proximal tubules of the kidney [89]. Two types of SGLT have been identified: SGLT1 and SGLT2. SGLT2 regulates about 90% of glucose reabsorption, with the remainder accomplished by SGLT1 [90].

SGLT2is selectively block SGLT2 activity on the proximal renal tubule, leading to the removal of excess glucose in the urine and ultimately resulting in lower blood glucose levels. Additionally, SGLT2is reduce glycosylated proteins, improve insulin sensitivity, and enhance beta cell function [91]. SGLT2is were first introduced as medications for managing diabetes. They were also shown to provide benefits, such as reducing body weight and decreasing blood pressure [92]. More critically, treatment with SGLT2is has been shown to reduce the risk of cardiovascular disease in patients with T2DM when compared with placebo treatment [93, 94]. There is much evidence to suggest that SGLT2is may have the potential to reduce inflammatory responses [95–98], myocardial fibrosis [95], insulin resistance [99, 100], and oxidative stress [101, 102], making them a promising treatment option for DHD. SGLT2is-related clinical studies are shown in Table 1; SGLT2is-related preclinical studies are shown in Table 2.

**Table 1:** Clinical studies of SGLT2is for the treatment of DM or DM-related diseases

Medicine	Generic Name	First Author	Year	Disease	Model	Findings	Ref.
SGLT2is	Dapagliflozin	Kato, E.T.	2019	T2DM/HFrEF	Human	Dapagliflozin reduced cardiovascular and all-cause mortality in patients with high rates of HFrEF and in patients with T2DM	[93]
		Furtado, R.H.M.	2019	T2DM/MI	Human	Dapagliflozin reduced risk of MACE and cardiovascular death/hospitalization for HF in patients with T2DM and prior MI	[94]
		McMurray, J.J.V.	2019	HFrEF	Human	Dapagliflozin reduced risk of worsening HF or death from cardiovascular disease in patients with HErEF	[103]
		Wiviott, S.D.	2019	T2DM	Human	Dapagliflozin reduced cardiovascular mortality and hospitalization for HF in patients with T2DM	[104]
	Empagliflozin	Zinman, B.	2015	T2DM	Human	Empagliflozin reduced incidence of major composite cardiovascular outcomes and death from any cause in patients with T2DM, at high risk for cardiovascular events	[105]
		Iannantuoni, F.	2019	T2DM	Human	Empagliflozin reduced inflammation and oxidative stress in patients with T2DM	[96]
	Canagliflozin	Neal, B.	2017	T2DM	Human	Canagliflozin reduced incidence of cardiovascular and renal events in patients with T2DM	[106]

HFrEF heart failure with reduced ejection fraction, MI myocardial infarction, MACE major adverse cardiovascular events

**Table 2:** Preclinical studies of SGLT2is for the treatment of DM or DM-related diseases

Medicine	Generic Name	First Author	Year	Disease	Model	Findings	Ref.
SGLT2is	Dapagliflozin	Lee, T.M.	2017	MI	Rat	Dapagliflozin attenuated myocardial fibrosis in rats after MI	[95]
		Terami, N.	2014	T2DM	Mouse	Dapagliflozin significantly reduced macrophage infiltration and expression of inflammation and oxidative stress genes in the kidneys of T2DM mice	[101]
		Ye, Y.	2017	DCM	Mouse	Dapagliflozin inhibited Nlrp3/ASC inflammasome activation and attenuated DCM in T2DM mice	[107]
	Empagliflozin	Xu, L.	2017	Obesity	Mouse	Empagliflozin improved insulin sensitivity	[99]
		Oelze, M.	2014	T1DM	Rat	Empagliflozin ameliorated oxidative stress and inflammatory response in T1DM rats	[102]
		Kern, M.	2016	DM	Mouse	Empagliflozin improved insulin sensitivity in DM mice	[108]
		Habibi, J.	2017	DM	Mouse	Empagliflozin improved cardiac diastolic function in a female mouse model of DM	[109]

MI myocardial infarction, ASC apoptosis-associated speck-like protein containing a CARD

### Glucagon-like peptide 1 receptor agonists (GLP1RAs)

Glucagon-like peptide 1 (GLP-1) is produced by the posttranslational proteolytic cleavage of proglucagon protein [110]. GLP-1 can enhance insulin secretion in a glucose-dependent manner by activating the GLP-1 receptor (GLP1R) that is highly expressed on islet β cells [111]. GLP1RAs, the drugs used to treat patients with diabetes, also provide benefits for patients with cardiovascular disease. Obesity and diabetes are both risk factors for cardiovascular disease. According to a study by Lazzaroni et al. [112], GLP1RAs significantly reduced body weight in patients with T2DM. A systematic review and meta-analysis of cardiovascular outcome trials of GLP1RAs showed that GLP1RA treatment reduced major adverse cardiovascular events (MACE) by 12% and reduced all-cause mortality by 12% [113]. Furthermore, in patients treated with GLP1RAs, HF admission rates were reduced by 9% [113]. However, in another meta-analysis, GLP1RAs had no significant effect on HF admission rates [114]. Thus, although the benefit of GLP1RAs for patients with cardiovascular disease has been established, the benefits are less certain for patients with HF. Because advanced DHD is characterized by HF [32, 115], GLP1RAs may have little effect on patients with advanced DHD. However, whether GLP1RAs are effective in treating patients with early DHD or preventing the development of DHD warrants further investigation.

Preclinical studies have shown that GLP1RAs have the potential to treat DHD. The GLP1RA liraglutide increases the myocardial glucose oxidation rate and alleviates DCM in C57BL/6J mice [116]. Furthermore, liraglutide-treated rats with DCM showed reduced inflammation, myocardial fibrosis, and apoptosis [117]. Researchers showed that using the GLP1RA exenatide to treat mice with DCM significantly improved serum B-type natriuretic peptide, myocardial fibrosis, myocardial lipid deposition, and echocardiographic parameters [7]. Other researchers showed that treatment with the novel oral GLP1RA oral hypoglycemic peptide 2 (OHP2) prevents DCM in rats by alleviating cardiac lipotoxicity-induced mitochondrial dysfunction [118].

GLP1RAs treatment of DCM may be closely related to their interactions with macrophages. Under pathological stress, macrophages contribute to an excessive inflammatory response, which leads to insulin resistance and diabetes. GLP1RAs can attenuate macrophage infiltration and inhibit the expression of IL-1β, IL-6, and TNF-α [119]. In addition, GLP1RAs can inhibit macrophage polarization to the M1 pro-inflammatory phenotype [120] and promote macrophage polarization to the M2 anti-inflammatory phenotype [121, 122]. The large amount of pro-inflammatory cytokines and chemokines in adipose tissue is a key factor contributing to insulin resistance in patients with T2DM [60, 123, 124]. GLP1RAs inhibit inflammatory mediators in adipose tissue and contribute to improved insulin sensitivity [125]. Moreover, GLP1RAs demonstrate a direct protective effect on the development of diabetes-associated myocardial fibrosis and diastolic dysfunction [126]. Taken together, GLP1RAs contribute to relieving DHD by modulating macrophage function. GLP1RAs-related clinical studies are shown in Table 3; GLP1RAs-related preclinical studies are shown in Table 4.

**Table 3:** Clinical studies of GLP1RAs for the treatment of DM or DM-related diseases

**Medicine**	**Generic Name**	**First Author**	**Year**	**Disease**	**Model**	**Findings**	**Ref.**
GLP1RAs	Dulaglutide	Gerstein, H.C.	2020	T2DM	Human	Long-term dulaglutide use might reduce clinically relevant ischemic stroke in people with T2DM	[127]
		Tuttolomondo, A.	2021	T2DM	Human	Positive effects on arterial stiffness and endothelial function indicators in patients with T2DM receiving conventional therapy with daily subcutaneous injections of 1.5 mg dulaglutide	[128]
	Liraglutide	Marso, S.P.	2016	T2DM	Human	The composite endpoint of death from cardiovascular causes, nonfatal myocardial infarction, or nonfatal stroke was significantly lower in T2DM patients at high cardiovascular risk	[129]
	Semaglutide	Husain, M.	2019	T2DM	Human	The cardiovascular risk profile of patients with T2DM taking oral semaglutide was not worse than those taking placebo	[130]
		Strain, W.D.	2022	T2DM	Human	Semaglutide treatment reduced stroke risk in patients with T2DM and higher cardiovascular risk compared with placebo treatment	[131]
	Efpeglenatide	Gerstein, H.C.	2021	T2DM	Human	Patients with T2DM who received weekly subcutaneous doses of 4 or 6 mg of efpeglenatide had a lower risk of cardiovascular events than those on placebo	[132]
	Albiglutide	Hernandez, A.F.	2018	T2DM	Human	In patients with T2DM and cardiovascular disease, albiglutide was superior to placebo with respect to major adverse cardiovascular events	[133]

**Table 4:** Preclinical studies of GLP1RAs for the treatment of DM or DM-related diseases

Medicine	Generic Name	First Author	Year	Disease	Model	Findings	Ref.
GLP1RAs	Liraglutide	Almutairi, M.	2021	DCM	Mouse	Liraglutide increased myocardial glucose oxidation and mitigated DCM	[116]
		Trang, N.N.	2021	DCM	Rat	Liraglutide reduced inflammation, myocardial fibrosis, and apoptosis in DCM rats	[117]
	Exenatide	Fang, P.	2023	DCM	Mouse	Exenatide significantly improved serum BNP, myocardial fibrosis, myocardial lipid deposition, and echocardiographic parameters in DCM mice	[7]
	OHP2	Qian, P.	2020	DCM	Rat	OHP2 prevented DCM by alleviating cardiac lipotoxicity-induced mitochondrial dysfunction	[118]
	Recombinant adenovirus producing GLP-1	Lee, Y.S.	2012	DM/ Obesity	Mouse	Recombinant adenovirus producing GLP-1 improved insulin sensitivity	[125]
	Exendin-4	Tate, M.	2016	DM	Mouse	Exendin-4 had a protective effect on the development of diabetes-related myocardial fibrosis and diastolic dysfunction	[126]

BNP brain natriuretic peptide, OHP2 oral hypoglycemic peptide 2

### Metformin

Metformin is a first-line medication for treating patients with T2DM [134] and works by decreasing the production of glucose in the liver and activating the adenosine monophosphate-activated protein kinase (AMPK). Because metformin activates AMPK, it may also induce the regression of myocardial hypertrophy [31]. In addition, metformin enhances insulin sensitivity by increasing the activity of insulin receptor tyrosine kinase, thereby promoting glycogen synthesis and improving the recruitment and activity of the glucose transporter 4 (GLUT4) [135]. In a mouse model of transverse aortic constriction (TAC)-induced HF, treatment with metformin attenuated myocardial fibrosis by inhibiting the TGFβ1-Smad3 signaling pathway [136]. In addition, clinical studies have shown that metformin treatment reduced the incidence of HF in patients with diabetes [137, 138]. Collectively, this evidence suggests that metformin has potential for use in the treatment of DHD.

Studies have shown that metformin provides benefits by its effects on macrophages. In human macrophages, treatment with metformin selectively inhibited the differentiation of human monocytes into pro-inflammatory M1 macrophages [139]. In addition, LPS stimulated M2 macrophages to produce ROS that are harmful to surrounding tissues, a process that was inhibited by the addition of metformin [139]. Interestingly, metformin reduces oxidative stress and inflammatory responses by inhibiting the differentiation of human monocytes into M1 macrophages and limiting macrophage ROS production through the activation of AMPK [139]. Metformin can also exert anti-inflammatory effects by modulating the AMPK/mTOR signaling pathway to inhibit activation of the NLRP3 inflammasome and favor macrophage polarization toward the M2 phenotype [140]. These findings suggest that metformin acts as a cardioprotective and anti-inflammatory agent by stimulating AMPK/autophagy and thus inhibiting the NLRP3 inflammasome, which is closely associated with macrophages in DHD [141].

HMGB1 is released by necrotic cells and is a potential target for the development of anti-inflammatory therapies [142]. Metformin was found to significantly reduce the inflammatory response of LPS-stimulated macrophages in mice (in vivo) and in RAW 264.7 cells (in vitro) by inhibiting HMGB1 secretion [143]. Guo et al. [144] showed that metformin alleviated olanzapine-induced insulin resistance by inhibiting macrophage infiltration and polarization-mediated inflammatory responses in white adipose tissue of rat epididymis. Cortés et al. [145] found that the anti-inflammatory and inhibitory effects of metformin on ROS were dependent on the expression of the plasticity factor ZEB1 in macrophages. Therefore, metformin may potentially be used to treat DHD. Metformin-related clinical studies are shown in Table 5; metformin-related preclinical studies are shown in Table 6.

**Table 5:** Clinical studies of metformin for the treatment of DM or DM-related diseases

Medicine	First Author	Year	Disease	Model	Findings	Ref.
Metformin	van der Aa, M.P.	2016	Obesity	Human	Long-term treatment of obese and insulin-resistant adolescents with metformin stabilized BMI and reduced insulin resistance compared with placebo	[134]
	Hippisley-Cox, J.	2016	T2DM	Human	Combination therapy with metformin and gliptins reduced risk of HF in patients with T2DM	[137]
	Panagiotopoulou, O.	2020	Obesity	Human	Children of obese mothers who were exposed prenatally to metformin compared with those who were exposed to placebo had lower central hemodynamic and cardiac diastolic indices	[146]
	Timmons, J.G.	2021	T1DM	Human	Metformin reduced carotid intima-media thickness in never-smoking patients with T1DM	[147]
	Eurich, D.T.	2013	DM/HF	Human	Metformin was safe for treating patients with DM combined with HF and did not increase the risk of lactic acidosis	[148]
	Bhansali, S.	2020	T2DM	Human	Metformin upregulated mitochondrial autophagy in patients with T2DM, resulting in improved mitochondrial morphology and function independent of its glucose-lowering effect	[149]

BMI body mass index

**Table 6:** Preclinical studies of metformin for the treatment of DM or DM-related diseases

Medicine	First Author	Year	Disease	Model	Findings	Ref.
Metformin	Xiao, H.	2010	HF	Mouse	Metformin attenuated myocardial fibrosis by inhibiting the TGFβ1-Smad3 signaling pathway	[136]
	Nassif, R.M.	2022	Inflammation	Mouse	Metformin inhibited ROS production by activating AMPK	[139]
	Yang, F.	2019	DCM	Mouse	Metformin activated AMPK, improving autophagy by inhibiting the mTOR pathway and alleviating focal death in DCM	[150]
	Tsoyi, K.	2011	Endotoxemia	Mouse	Metformin improved survival in a mouse model of lethal endotoxemia by inhibiting HMGB1 release	[151]

mTOR mammalian target of rapamycin

### Renin-angiotensin-aldosterone system inhibitors (RAASis)

Activation of the RAAS in patients with diabetes leads to inflammation, cardiac fibrosis, and oxidative stress, all of which contribute to cardiac remodeling and can be reversed or prevented by RAAS blockade [152, 153]. RAASis include angiotensin-converting enzyme inhibitors (ACEis), angiotensin II receptor blockers (ARBs), renin inhibitors, and mineralocorticoid receptor antagonists (MRAs) [154]. ACEis reduce cardiovascular disease incidence and all-cause mortality and increase cellular insulin sensitivity in patients with diabetes [155]. In a rat model of ischemic cardiomyopathy, the ARB valsartan attenuates TLR activity, inhibits NFκB activity, and reduces circulating cytokine levels [156]. Candesartan, another ARB, ameliorates abnormal local calcium release from cardiomyocytes in the atrial tissue of rats with DCM [157]. Renin inhibitors, such as aliskiren, improve left ventricular hypertrophy and end-systolic volume in patients with diabetes [158, 159]. These studies suggest that RAASis have the potential to treat DHD. The activation of RAAS contributes to the infiltration of macrophages, and the antagonistic macrophage mineralocorticoid receptors have a significant protective effect on cardiovascular remodeling [160]. Activated macrophages produce angiotensin-converting enzyme (ACE), which induces local expression of angiotensin II (Ang II). The ability of ACEis to reduce left ventricular mass and ameliorate myocardial fibrosis suggests a direct link between macrophages, macrophage-derived ACE, myofibroblasts, and left ventricular remodeling [161]. Studies have shown that aldosterone induces galectin-3 expression in macrophages and vascular endothelial cells that leads to vascular and cardiac fibrosis, implying a correlation between RAAS and macrophages [162]. In addition, adipose-infiltrating macrophages have been shown to secrete pro-inflammatory cytokines, such as IL-6 and TNF-α, which trigger activation of the RAAS [163]. In summary, a positive-feedback mechanism may exist between macrophages and the RAAS, leading to mutual activation; therefore, RAASis may indirectly inhibit macrophage hyperactivation, resulting in a therapeutic effect on DHD. RAASis-related clinical studies are shown in Table 7; RAASis-related preclinical studies are shown in Table 8.

**Table 7:** Clinical studies of RAASis for the treatment of DM or DM-related diseases

**Medicine**	**Generic Name**	**First Author**	**Year**	**Disease**	**Model**	**Findings**	**Ref.**
RAASis	Aliskiren, Losartan	Solomon, S.D.	2009	Hypertension	Human	Aliskiren and losartan attenuated myocardial end-organ damage effectively	[158]
	Aliskiren	Shah, A.M.	2012	DM/MI	Human	Aliskiren improved left ventricular hypertrophy and end-systolic volume in patients with DM	[159]
	Captopril	Hansson, L.	1999	Hypertension/DM	Human	Captopril reduced the propensity to develop T2DM by 11% in hypertensive patients	[164]
	Ramipril	Yusuf, S.	2000	Hypertension/DM	Human	Ramipril reduced the propensity to develop T2DM by 34% in hypertensive patients	[165]
	ARBs	Lambers Heerspink, H.J.	2012	T2DM	Human	Moderation of dietary sodium potentiated the renal and cardiovascular protective effects of ARBs	[166]

**Table 8:** Preclinical studies of RAASis for the treatment of DM or DM-related diseases

Medicine	Generic Name	First Author	Year	Disease	Model	Findings	Ref.
RAASis	Lisinopril	Fiordaliso, F.	2006	DM	Rat	Lisinopril reduced cardiovascular oxidative stress in DM rats	[152]
	Olmesartan	Matsusaka, H.	2006	DM/MI	Mouse	Olmesartan improved left ventricular remodeling and failure after MI in DM mice	[153]
	Valsartan	Yang, J.	2009	MIRI	Rat	Valsartan prevented myocardial ischemia-reperfusion injury in rats via TLR4/NF-κB signaling pathway	[156]
	Candesartan	Yaras, N.	2007	DCM	Rat	Candesartan protected cardiomyocytes from adverse effects associated with abnormal Ca2^+^ release mechanisms in DCM rats	[157]

MIRI myocardial ischemia-reperfusion injury

### β2-adrenergic receptor agonists (β2ARAs)

G protein–coupled receptors (GPCRs) are important proteins that mediate most cellular responses to external stimuli [167]. Adrenergic receptors (ARs), belonging to the GPCR family, contain both α and β subtypes. β ARs can be further subdivided into β1, β2, and β3 ARs, which are located on the surface of effector cells. β2AR has been extensively studied and is found predominantly in human smooth muscle, where it regulates a variety of physiologic processes [168, 169]. Many β2ARAs have been developed. Because β2ARAs induce relaxation of airway smooth muscle, they are used to treat various respiratory diseases, particularly asthma and chronic obstructive pulmonary disease (COPD) [170]. However, many potential uses for β2ARAs remain to be explored, such as in the treatment of DHD.

In a screening of 1040 compounds with anti-inflammatory effects in rat bone marrow macrophages, researchers identified β2ARA as the most potent compound in inhibiting NFκB-dependent pro-inflammatory TNF-α production by macrophages [171]. Subsequently, they found that β2ARAs inhibited TNF-α production in peripheral blood mononuclear cells of streptozotocin (STZ)-induced diabetic rats [171]. To elucidate the mechanism, they exposed human monocytic leukemia cells and bone marrow macrophages to a high-glucose environment. High glucose reduced the expression of β-arrestin2, a negative regulator of NFκB activation, and its interaction with IκBα, subsequently enhancing the phosphorylation of IκBα and the activation of NFκB. The addition of β2ARAs enhanced the expression of β-arrestin2 and its interaction with IκBα, which led to the downregulation of NFκB. siRNA specific for β-arrestin2 reversed the β2ARA-mediated inhibition of NFκB activation and inflammatory cytokine production. In addition, Zucker diabetic fatty (ZDF) rats treated with the β2ARA salbutamol for 12 weeks showed attenuated monocyte activation, as well as pro-inflammatory and profibrotic responses in the kidneys and heart [172]. Several other studies [171, 173] have shown beneficial effects of β2ARAs in treating DM and its complications. Notably, inhibiting macrophage activation and cardiomyopathy progression with β2ARAs only occurs under hyperglycemic conditions and not normoglycemic conditions [174]. In summary, β2ARAs may be promising drugs for treating DHD. β2ARAs-related clinical studies are shown in Table 9; β2ARAs-related preclinical studies are shown in Table 10.

**Table 9:** Clinical studies of β2ARAs for the treatment of DM or DM-related diseases

Medicine	Generic Name	First Author	Year	Disease	Model	Findings	Ref.
β2ARAs	Zinterol	Kaumann, A.	1999	HF	Human	Zinterol improved cardiac diastolic function	[175]
	Caveolin-3	Gong, J.	2020	DM/MIRI	Human	Caveolin-3 protected diabetic hearts against I/R damage through the β2AR, cAMP/PKA, and BDNF/TrkB signaling pathways	[176]

BDNF brain-derived neurotrophic factor, cAMP cyclic adenosine monophosphate, I/R ischemia reperfusion, PKA protein kinase A, TrkB tropomyosin-related kinase receptor type B

**Table 10:** Preclinical studies of β2ARAs in DM or DM-related diseases

Medicine	Target/Drug Name	First Author	Year	Disease	Model	Findings	Ref.
β2ARAs	β2ARA	Noh, H.	2017	DM	Rat	β2ARA attenuated monocyte activation and pro-inflammatory and pro-fibrotic responses in the kidney and heart	[172]
	Thalidomide	Zhang, H.	2018	DM	Rat	Thalidomide may have therapeutic potential for diabetic kidney injury through an anti-inflammatory pathway	[173]
	Leukocyte-expressed β2AR	Grisanti, L.A.	2016	MI	Rat	Leukocyte-expressed β2AR played an important role in regulating the early inflammatory repair response to acute myocardial injury by promoting cardiac leukocyte infiltration	[174]
	Clenbuterol	van Beek, S.M.M.	2021	T2DM	Mouse	Clenbuterol improved whole-body glucose homeostasis	[177]

### Potential therapeutic strategy

#### Targeting MicroRNAs (MiRNAs)

MicroRNAs (miRNAs) are small RNA molecules that are important regulators of different cellular processes. MiRNAs control gene expression at the post-transcriptional level by disassembling or inhibiting the translation of target messenger RNAs (mRNAs) by binding to their 3’-untranslated region (3’UTR) [178].

MiRNAs play important roles in many aspects of macrophage biology, particularly in immune cells, such as monocytes and macrophages [179]. They regulate polarization, differentiation, inflammation, and phagocytosis [180]. For example, miR-720 and miR-127 promote M1 macrophage polarization and suppress M2 polarization by targeting GATA binding protein 3 (GATA3) and B-cell lymphoma 6 (BCL6), respectively, which are critical for M2 polarization [181, 182]. MiR-155 promotes M1 polarization. Both gain-of-function and loss-of-function studies in vivo have shown that miR-155 is necessary for the typical development of the macrophage inflammatory state [183]. Abdominal macrophages that overexpress miR-146a showed an increase in M2-type marker genes (e.g., cluster of differentiation 206 (CD206), arginase 1 (ARG1), C-C motif chemokine ligand 22 (CCL22), and CCL17) and a decrease in M1-type phenotypic markers (e.g., inducible nitric oxide synthase, IL-12, IL-6, TNF, and CD86) [184], indicating that miR-146a may have anti-inflammatory effects.

MiRNAs have been shown to be involved in the process of developing DCM. Myocyte enhancer factor 2 C (MEF2C) is a key transcription factor in promoting cardiomyocyte hypertrophy [185]. In mice and rats with DCM, both miR-133a and miR-373 are involved in MEF2C signaling, leading to cardiomyocyte hypertrophy and mediating cardiac fibrosis through the activation of the p300 gene [185, 186]. In addition, miR-208a [187] and miR-451 [188] are involved in cardiomyocyte hypertrophy. Liu et al. [189] showed that miR-21 levels were significantly elevated in cardiac fibroblasts treated with high glucose, leading to increased collagen synthesis and elevated phosphorylated p38 mitogen-activated protein kinase (MAPK). In addition, inhibiting miR-21 by blocking the activation of the p38 signaling pathway reduced fibrosis, suggesting that miR-21 plays a critical role in DCM. Moreover, miRNAs are associated with oxidative stress in rats with DCM. The significant downregulation of miR-499, miR-1, and miR-133 was observed in high glucose–treated cardiomyocytes, which was reversed by treatment with the antioxidant N-acetylcysteine, suggesting that the downregulation of these miRNAs in the diabetic heart is caused by oxidative stress [190].

Perhaps more importantly, miRNAs modulate macrophage polarization that occurs during the development of DCM. Wang and colleagues showed that miR-657 regulates inflammation and insulin resistance in patients with diabetes by targeting the *FAM46C* gene to promote macrophage polarization toward M1 [191]. A study discovered that miR-223, a significant regulator of macrophage polarization, suppresses macrophage activation toward a pro-inflammatory phenotype by inhibiting *Pknox1*. This prevents high-fat-diet-induced adipose tissue inflammatory response and systemic insulin resistance in mice [192]. Thus, miRNAs may control the inflammatory response and insulin resistance by regulating macrophage polarization. Furthermore, miRNAs may be involved in the pathogenesis of DCM by regulating genes related to cardiomyocyte hypertrophy, oxidative stress, and cardiac fibrosis. Together, these findings suggest that targeting miRNAs may be a novel therapeutic strategy for the treatment of DCM. MicroRNAs-related preclinical studies are shown in Table 11.

**Table 11:** Preclinical studies of microRNAs in DM or DM-related cardiovascular diseases

MicroRNAs	First Author	Year	Disease	Model	Findings	Ref.
MiR-471-3p	Liu, G.	2021	DCM	Mouse	The development of DCM was related to AGE-induced macrophage polarized to M1 type through a mechanism involving the miR-471-3p/SIRT1 pathway	[71]
MiR-155	Fitzsimons, S.	2020	Atherosclerosis	Mouse	MiR-155 suppressed anti-inflammatory signaling in macrophages, and it has potential as a prognostic indicator and a therapeutic target	[193]
	Jia, C.	2017	DCM	Mouse	Therapeutically reducing miR155 in macrophages by AuNP can serve as a promising strategy in improving cardiac function	[194]
MiR-21/99a/146b/378a, MiR-33	Phu, T.A.	2022	DM	Mouse	THP1-IL4-exo polarized primary macrophages to an anti-inflammatory phenotype and reprogramed their energy metabolism by increasing levels of miR-21/99a/146b/378a while reducing miR-33	[195]
MiR-99a/146b/378a	Bouchareychas, L.	2020	Atherosclerosis	Mouse	BMDM-exo contain anti-inflammatory microRNA-99a/146b/378a. These exosomal microRNAs suppressed inflammation by targeting NF-κB and TNF-α signaling and fostering M2 polarization in recipient macrophages	[196]
MiR-27-3p	Li, J.	2023	T2DM	Mouse	Inactivation of miR-27-3p induced by M1 exosomes prevented T2DM development in high-fat-diet-fed mice	[197]
MiR-330-5p	Sun, J.	2018	T2DM	Mouse	MiR-330-5p/Tim-3 axis downregulated insulin resistance in diabetes, probably through enhancing the M2 polarization of macrophage	[198]
MiR-32	Cao, J.	2022	T2DM, Vascular Calcification	Mouse	Extracellular vesicle miR-32 derived from macrophage promoted arterial calcification in mice with T2DM via inhibiting VSMC autophagy	[199]
MiR-92a	Chang, Y.	2019	Atherosclerosis	Mouse	Extracellular miR-92a can be transported to macrophages through extracellular vesicles to regulate KLF4 levels, thus leading to the atheroprone phenotypes of macrophage	[200]
MiR-130b	Zhang, M.	2016	T2DM	Mouse	MiR-130b was a novel regulator of macrophage polarization and contributed to adipose tissue inflammation and insulin tolerance	[201]
MiR-126	Suresh Babu, S.	2016	DM	Mouse	In vitro efferocytosis of ACM was impaired in macrophages from diabetic mice, and miR-126 overexpression rescued diabetes-induced impairment in efferocytosis of ACM	[202]

ACM apoptotic cardiomyocytes, AuNP gold nanoparticle, BMDM-exo exosomes produced by naive bone marrow-derived macrophages, KLF4 Krüppel-like factor 4, SIRT1 silent information regulator 1, THP1-IL4-exo human THP-1 macrophages exposed to IL-4 as a source of exosomes, Tim-3 T cell immunoglobulin domain and mucin domain-3, VSMC vascular smooth muscle cell

#### Melatonin

Melatonin, an endogenous indoleamine hormone with potential free radical scavenging ability, is synthesized and secreted by the pineal gland in mammals and is primarily involved in physiological activities associated with the light-dark cycle [203]. Because of their wide distribution in the body, melatonin receptors exhibit a variety of biological activities beyond antioxidant activity, such as anti-inflammatory effects [204] and regulation of insulin secretion [205]. Fiorina et al. [206] showed that changes in melatonin secretion are related to the immune status of the body. In addition, melatonin may protect against cardiac complications in patients with DM by attenuating apoptotic pathways and addressing the inflammatory response and ROS burden by promoting macrophage polarization toward an anti-inflammatory state [207]. Further studies on melatonin-targeted regulation of macrophages in DHD should be explored.

## Conclusion

**Fig. 3:**
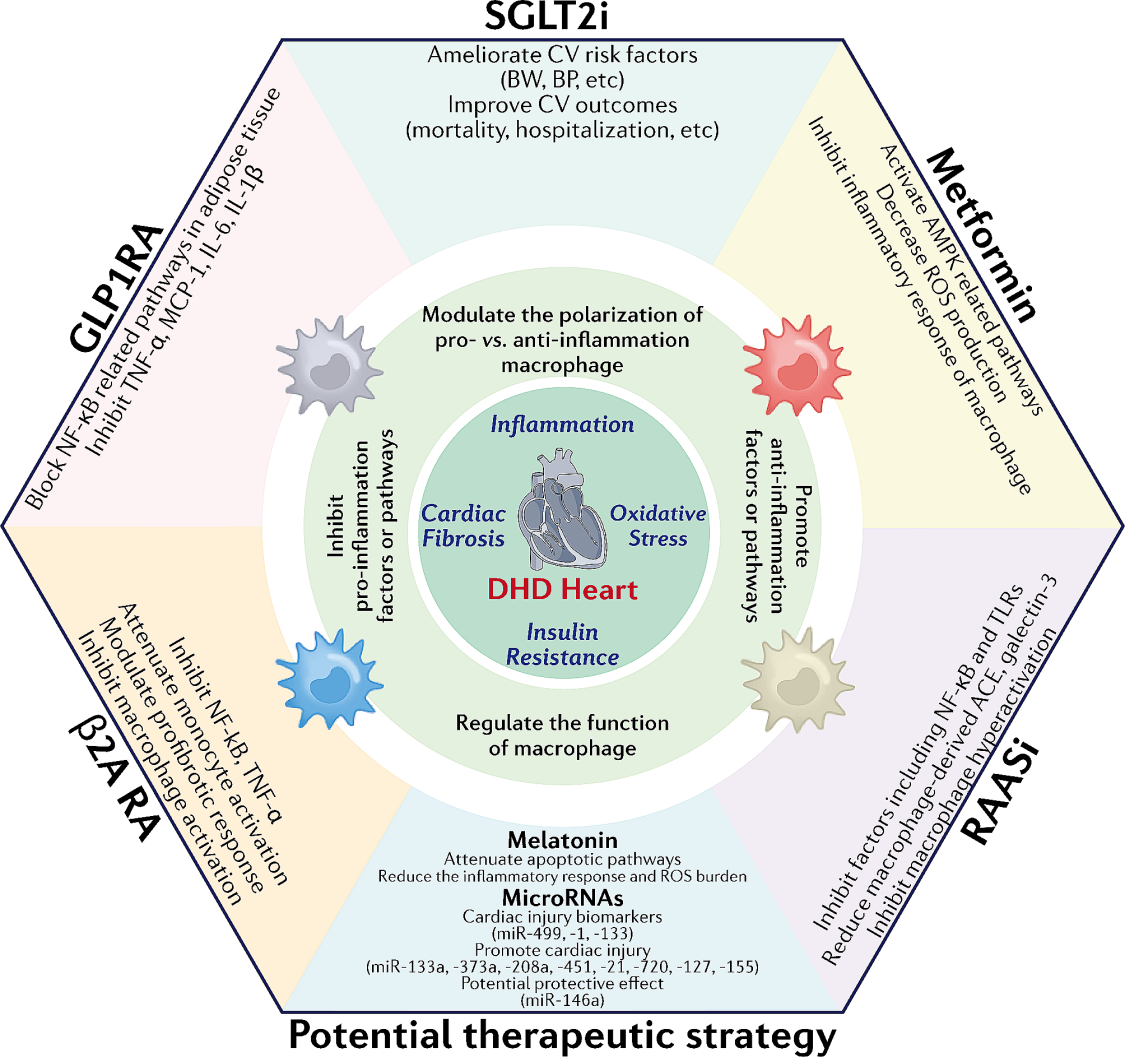
Summary schematic. Inflammation, myocardial fibrosis, insulin resistance, and oxidative stress are the four core mechanisms leading to DHD, and macrophages may play an essential role in these mechanisms. Five types of drugs—SGLT2is, GLP1RAs, metformin, RAASis, and β2ARAs—may have therapeutic potential by regulating macrophages. MiRNAs, which also regulate macrophages, may be involved in the pathogenesis of DHD and may also be potential targets for the treatment of DHD. In addition, melatonin is a promising research direction for treating DHD. Abbreviations: ACE: angiotensin-converting enzyme, AMPK: adenosine monophosphate-activated protein kinase, BW: body weight, BP: blood pressure, CV: cardiovascular, IL: interleukin, MCP-1: monocyte chemoattractant protein-1, NF-κB: nuclear factor kappa-B, ROS: reactive oxygen species, TLR: toll-like receptor, TNF-α: tumor necrosis factor-α

DHD is a serious complication of DM, posing a significant global health burden. Decades have passed since the discovery of DHD, but limited progress has been made in elucidating the mechanisms and identifying the therapeutic targets of DHD. Macrophages are crucial immune cells that play an indispensable role in maintaining normal physiological functions and resisting diseases. However, their role in the development of DHD has rarely been studied. In this review, we emphasized the significant role of macrophages in the pathogenesis of DHD. Macrophages play a major role in the development of DHD through mechanisms that primarily cause cardiac inflammation, insulin resistance, myocardial fibrosis, and oxidative stress. Additionally, the body’s hyperglycemic environment induces macrophage-trained immunity, which further contributes to the development of DHD. Finally, we present five classes of drugs that may have therapeutic effects on DHD by modulating macrophage function: SGLT2is, GLP1RAs, metformin, RAASis, and β2ARAs. Additionally, miRNAs may be a novel therapeutic for treating DHD, capable of targeting and modulating macrophages. In summary **(**Fig. 3**)**, macrophages hold great promise as therapeutic targets for DHD and have opened an exciting avenue for developing novel treatments for DHD.

## Data Availability

Not applicable.

## Abbreviations

ACE: Angiotensin-converting enzyme
ACEi: Angiotensin-converting enzyme inhibitor
AGE: Advanced glycation end product
AMPK: Adenosine monophosphate-activated protein kinase
Ang: Angiotensin
AR: Adrenergic receptor
ARA: Adrenergic receptor agonist
ARB: Angiotensin II receptor blocker
ARG: Arginase
ATM: Adipose tissue macrophage
BCL: B-cell lymphoma
BMDM: Bone marrow-derived macrophage
CAD: Coronary artery disease
CCR: C-C motif chemokine receptor
CCL: C-C motif chemokine ligand
CD: Cluster of differentiation
COPD: Chronic obstructive pulmonary disease
DAMP: Danger-associated molecular pattern
DCM: Diabetic cardiomyopathy
DHD: Diabetic heart disease
DM: Diabetes mellitus
ECM: Extracellular matrix
GAPDH: Glyceraldehyde-3-phosphate dehydrogenase
GLP1RA: Glucagon-like peptide 1 receptor agonist
GLUT: Glucose transporter
GPCR: G protein–coupled receptor
HbA1c: Hemoglobin A1c
HF: Heart failure
HFrEF: Heart failure with reduced ejection fraction
HMGB: High mobility group box
IKK: Inhibitor kappa B kinase
IL: Interleukin
INF: Interferon
JNK: c-Jun N-terminal kinase
MACE: Major adverse cardiovascular event
MAPK: Mitogen-activated protein kinase
MCP: Monocyte chemoattractant protein
M-CSF: Macrophage colony-stimulating factor
MEF: Myocyte enhancer factor
MRA: Mineralocorticoid receptor antagonist
NFκB: Nuclear factor kappa B
NLRP: NLR family pyrin domain-containing protein
NOX: Nicotinamide adenine dinucleotide phosphate oxidase
OHP: Oral hypoglycemic peptide
RAAS: Renin-angiotensin-aldosterone system
ROS: Reactive oxygen species
SGLT: Sodium-glucose cotransporter
TAC: Transverse aortic constriction
Th: T helper
TLR: Toll-like receptor
TNF: Tumor necrosis factor
3'UTR: 3'-untranslated region
ZDF: Zucker diabetic fatty
