# Correction: Therapeutic strategies targeting mechanisms of macrophages in diabetic heart disease

**DOI:** 10.1186/s12933-024-02300-4

**Published:** 2024-06-29

**Authors:** Chaoyue Zhang, Yunke Shi, Changzhi Liu, Shivon Mirza Sudesh, Zhao Hu, Pengyang Li, Qi Liu, Yiming Ma, Ao Shi, Hongyan Cai

**Affiliations:** 1 Cardiovascular Clinical Medical Center, The First Affiliated Hospital of Kunming Medical University, Kunming, China; 2 Department of Cardiovascular Surgery, The First Affiliated Hospital of Kunming Medical University, Kunming, China; 3 Faculty of Medicine, St. George University of London, London, UK; 4 University of Nicosia Medical School, University of Nicosia, Nicosia, Cyprus; 5 Department of Geriatric Cardiology, The First Affiliated Hospital of Kunming Medical University, Kunming, China; 6 Division of Cardiology, Pauley Heart Center, Virginia Commonwealth University, Richmond, VA USA; 7 Wafic Said Molecular Cardiology Research Laboratory, The Texas Heart Institute, Houston, TX USA

**Correction: Cardiovascular Diabetology (2024) 23:169** 10.1186/s12933-024-02273-4

Following publication of the original article [1], the author reported the errors in Tables 3 and 7. The entire content of the tables have been incorrectly published. The corrected Tables 3 and 7 are given below:

**Table 3:** Clinical studies of GLP1RAs for the treatment of DM or DM-related diseases

Medicine	Generic Name	First Author	Year	Disease	Model	Findings	Ref.
GLP1RAs	Dulaglutide	Gerstein, H.C.	2020	T2DM	Human	Long-term dulaglutide use might reduce clinically relevant ischemic stroke in people with T2DM	[127]
		Tuttolomondo, A.	2021	T2DM	Human	Positive effects on arterial stiffness and endothelial function indicators in patients with T2DM receiving conventional therapy with daily subcutaneous injections of 1.5 mg dulaglutide	[128]
	Liraglutide	Marso, S.P.	2016	T2DM	Human	The composite endpoint of death from cardiovascular causes, nonfatal myocardial infarction, or nonfatal stroke was significantly lower in T2DM patients at high cardiovascular risk	[129]
	Semaglutide	Husain, M.	2019	T2DM	Human	The cardiovascular risk profile of patients with T2DM taking oral semaglutide was not worse than those taking placebo	[130]
		Strain, W.D.	2022	T2DM	Human	Semaglutide treatment reduced stroke risk in patients with T2DM and higher cardiovascular risk compared with placebo treatment	[131]
	Efpeglenatide	Gerstein, H.C.	2021	T2DM	Human	Patients with T2DM who received weekly subcutaneous doses of 4 or 6 mg of efpeglenatide had a lower risk of cardiovascular events than those on placebo	[132]
	Albiglutide	Hernandez, A.F.	2018	T2DM	Human	In patients with T2DM and cardiovascular disease, albiglutide was superior to placebo with respect to major adverse cardiovascular events	[133]

**Table 7:** Clinical studies of RAASis for the treatment of DM or DM-related diseases

Medicine	Generic Name	First Author	Year	Disease	Model	Findings	Ref.
RAASis	Aliskiren, Losartan	Solomon, S.D.	2009	Hypertension	Human	Aliskiren and losartan attenuated myocardial end-organ damage effectively	[158]
	Aliskiren	Shah, A.M.	2012	DM/MI	Human	Aliskiren improved left ventricular hypertrophy and end-systolic volume in patients with DM	[159]
	Captopril	Hansson, L.	1999	Hypertension/DM	Human	Captopril reduced the propensity to develop T2DM by 11% in hypertensive patients	[164]
	Ramipril	Yusuf, S.	2000	Hypertension/DM	Human	Ramipril reduced the propensity to develop T2DM by 34% in hypertensive patients	[165]
	ARBs	Lambers Heerspink, H.J.	2012	T2DM	Human	Moderation of dietary sodium potentiated the renal and cardiovascular protective effects of ARBs	[166]

The original article has been corrected.
